# Supplementary material for: Systematics of Thraupis (Aves, Passeriformes) reveals an extensive hybrid zone between T. episcopus (Blue-gray Tanager) and T. sayaca (Sayaca Tanager)
Source: PLoS One. 2022 Oct 5;17(10):e0270892. doi: 10.1371/journal.pone.0270892 (PMC9534438; doi:10.1371/journal.pone.0270892)
Supplement: S1 Text — Abstract in Spanish. (PDF) [file pone.0270892.s001.pdf]

Resumen

El género *Thraupis* (Thraupidae), en la actualidad, está conformado por un total de siete especies de aves neotropicales, todas abundantes y communes dentro de sus zonas de distribución. Sin embargo, no existe una hipótesis filogenética con un amplio muestreo intraespecífico para el grupo. Debido a esto, los límites actuales de especie continúan siendo difusos. Con este fin, obtuvimos secuencias para dos marcadores mitocondriales (ND2, cyt-b) y tres marcadores nucleares no codificantes (TGFB2, MUSK y βF5) de 118 individuos con especímenes asociados en colecciones. Realizamos análisis de estructura genética, árbol coalescente de especies calibrado con un reloj molecular. Integramos los resultados moleculares con datos morfométricos y de coloración tomados de 1003 especimenes de museo con el objetivo de evaluar los límites de especie en *Thraupis*. Nuestros resultados confirman que *Thraupis* es un grupo monofilético que se originó en el Mioceno tardío y diversificó durante el Pleistoceno temprano. Encontramos que *Thraupis glaucocolpa* es la especie hermana de todas las otras especies en el género y a continuación *T. cyanoptera* de las cinco faltantes. Descubrimos una estructura genética en *Thraupis episcopus* que es congruente con patrones geográficos y de variación fenotípica en la especie. El primero de los grupos en *T. episcopus* se distribuye al este de los Andes y es diagnostivable por el parche blanco en las coberteras menores y medias del ala. El segundo grupo tiene un parche azul en la misa zona del ala y se distribuye principalmente al occidente de los Andes orientales. Finalmente, presentamos evidencia de hibridación y flujo genético entre diferentes taxones del género y a diferentes niveles taxonómicos, y discutimos su implicación en la clasificación taxonómica del grupo.
