# Supplementary figures and images for: Systematics of Thraupis (Aves, Passeriformes) reveals an extensive hybrid zone between T. episcopus (Blue-gray Tanager) and T. sayaca (Sayaca Tanager)
Source: PLoS One. 2022 Oct 5;17(10):e0270892. doi: 10.1371/journal.pone.0270892 (PMC9534438; doi:10.1371/journal.pone.0270892)

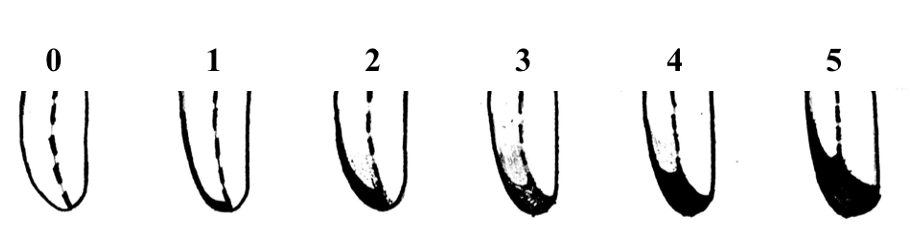

Supplement: S1 Fig — Illustration showing the difference of amount of white. Dash line denotes the main rachis of the greater coverts and the black area the amount of white on the feather. Values of 0 represent no white and 5 the maximum amount of white. (PNG) [file pone.0270892.s002.png]

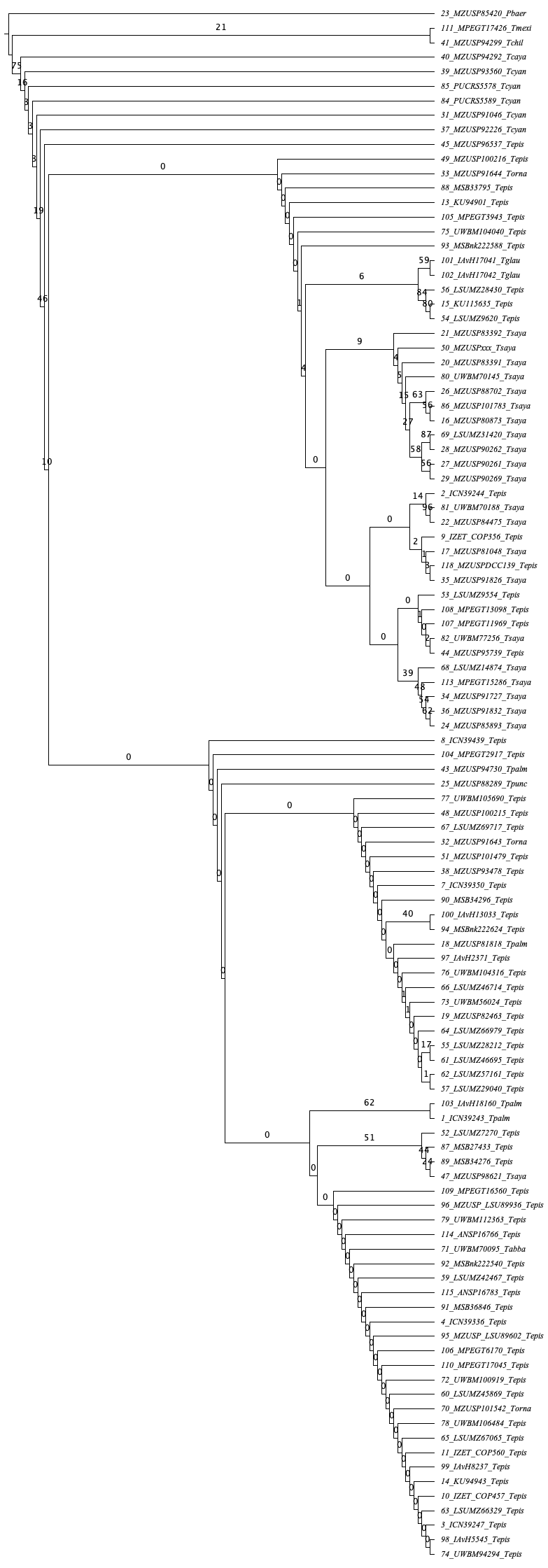

Supplement: S2 Fig — Raw tree files at figshare.com–S2 folder. (PNG) [file pone.0270892.s003.png]

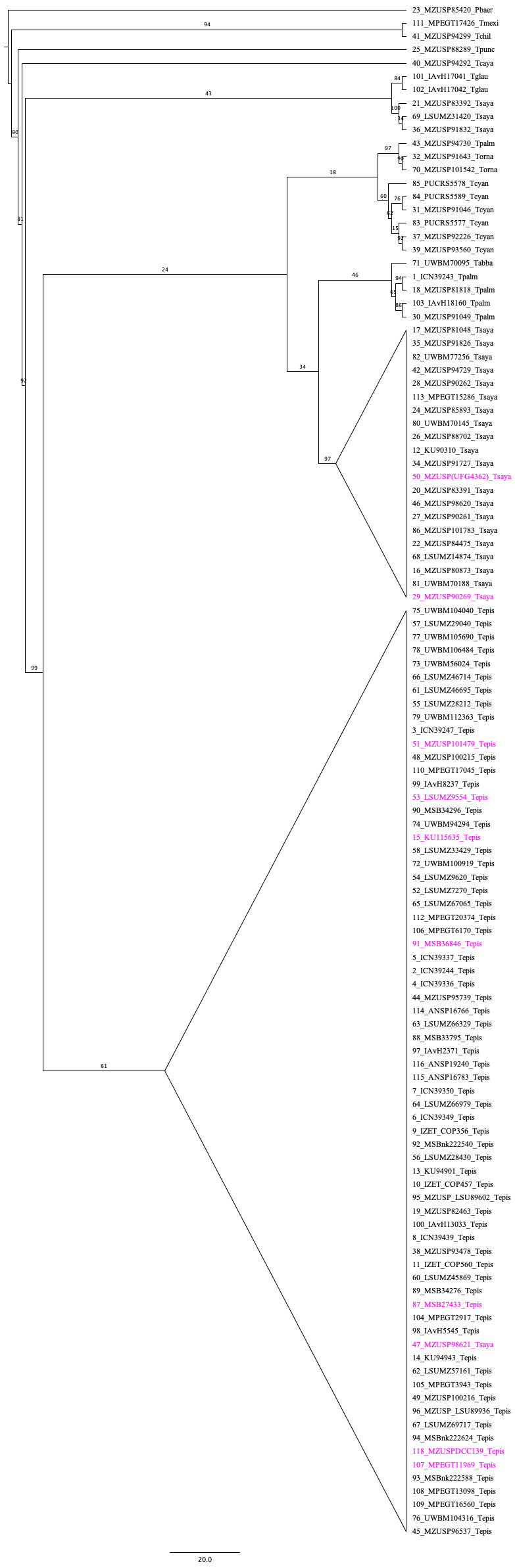

Supplement: S3 Fig — Raw tree files at figshare.com–S2 folder. (PNG) [file pone.0270892.s004.png]

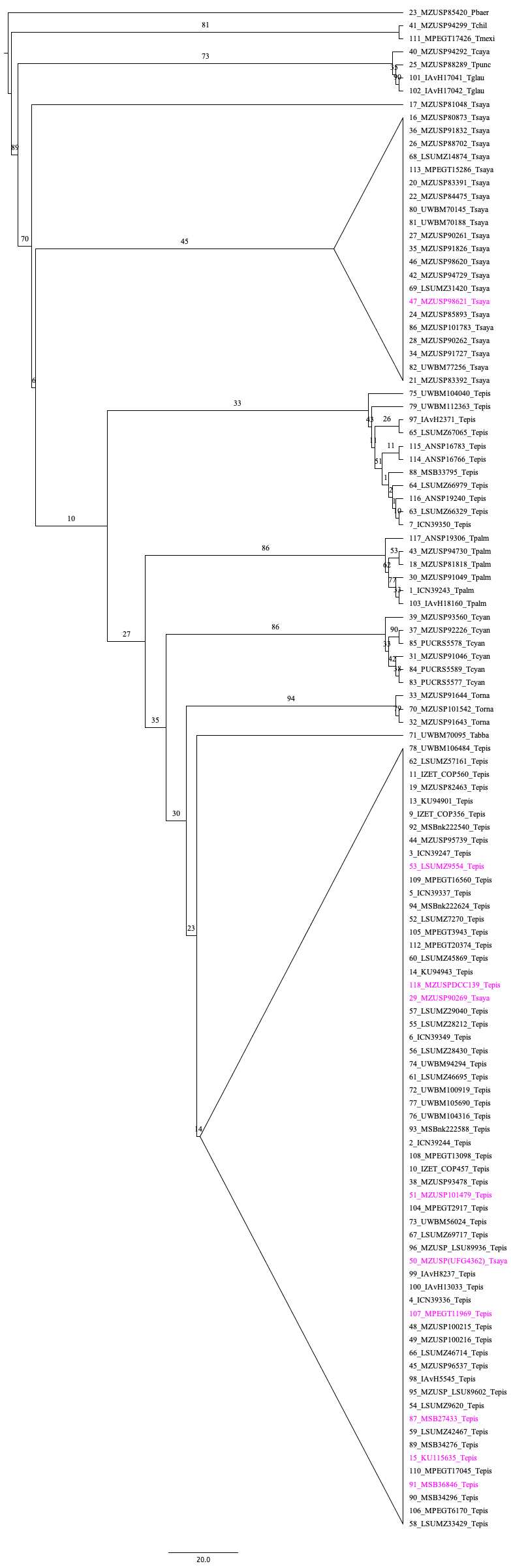

Supplement: S4 Fig — figshare.com–S2 folder. (PNG) [file pone.0270892.s005.png]

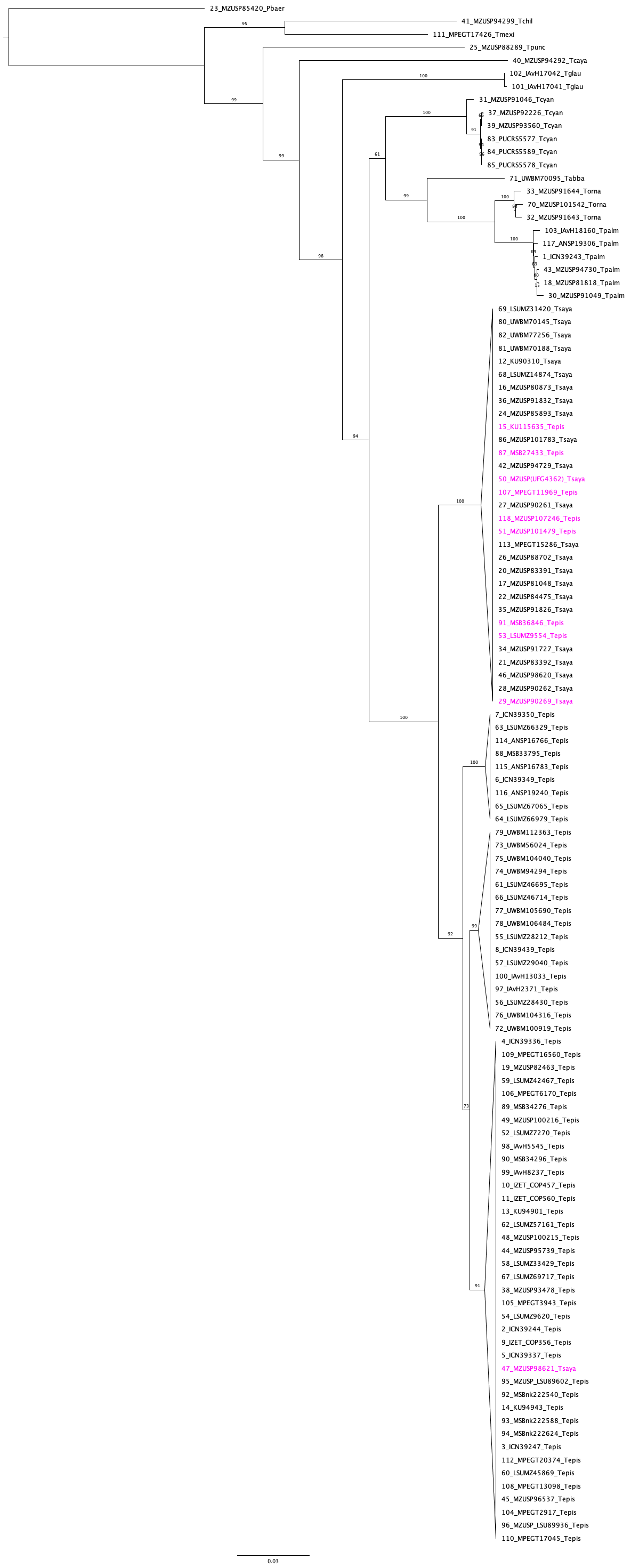

Supplement: S5 Fig — (PNG) [file pone.0270892.s006.PNG]

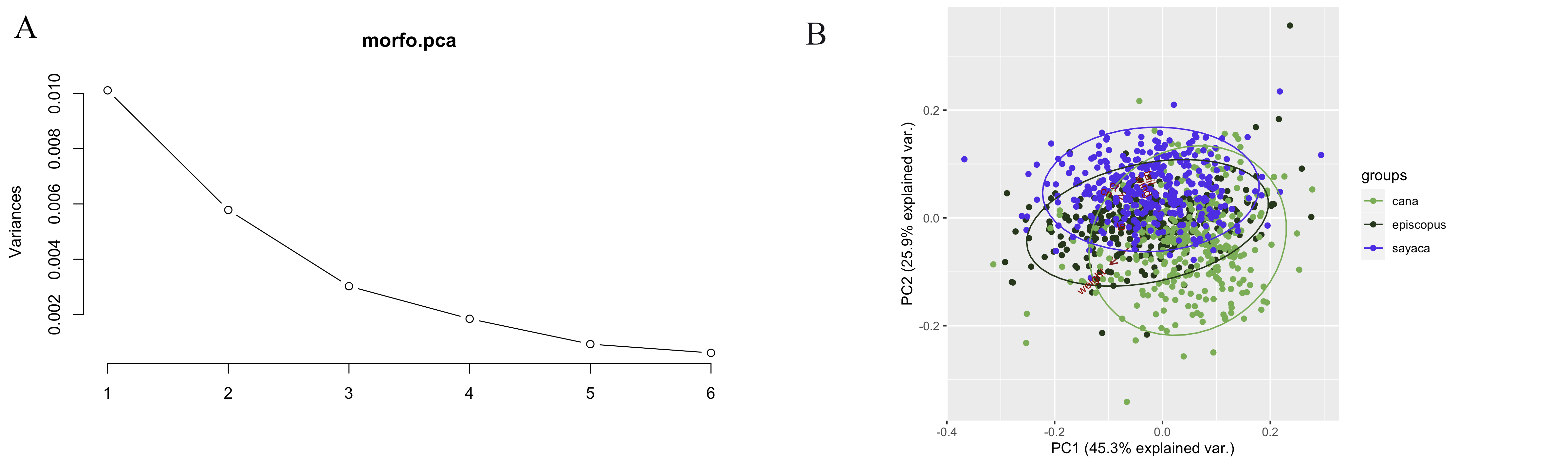

Supplement: S6 Fig — (A) Loadings of the PCA. (B) PCA of the three main groups recovered in genetic analyses. The names episcopus, cana and sayaca were given based on nomenclatural priority in each group. All comprise more than one named subspecies (see main S1 Text). (PNG) [file pone.0270892.s007.png]

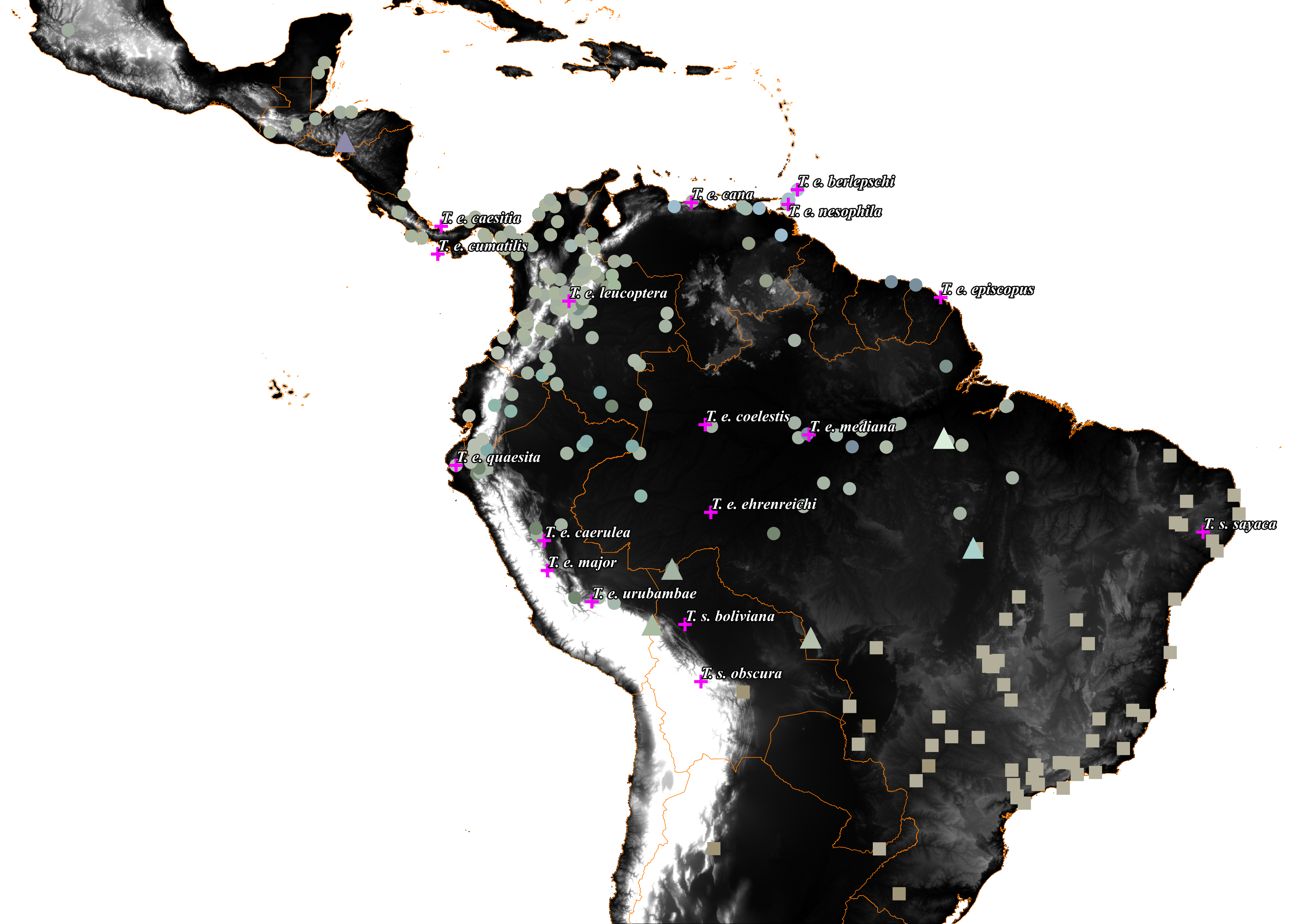

Supplement: S7 Fig — Each symbol represents a photographed specimen of T. episcopus (circles), T. sayaca (squares) or intermediate specimens (triangles). Symbol colors represent the actual color of the chest, as extracted from photography with the HTML code. Pink crosses denote the type locality of each subspecies. Raw pictures available at figshare.com—S1 folder. (PNG) [file pone.0270892.s008.png]

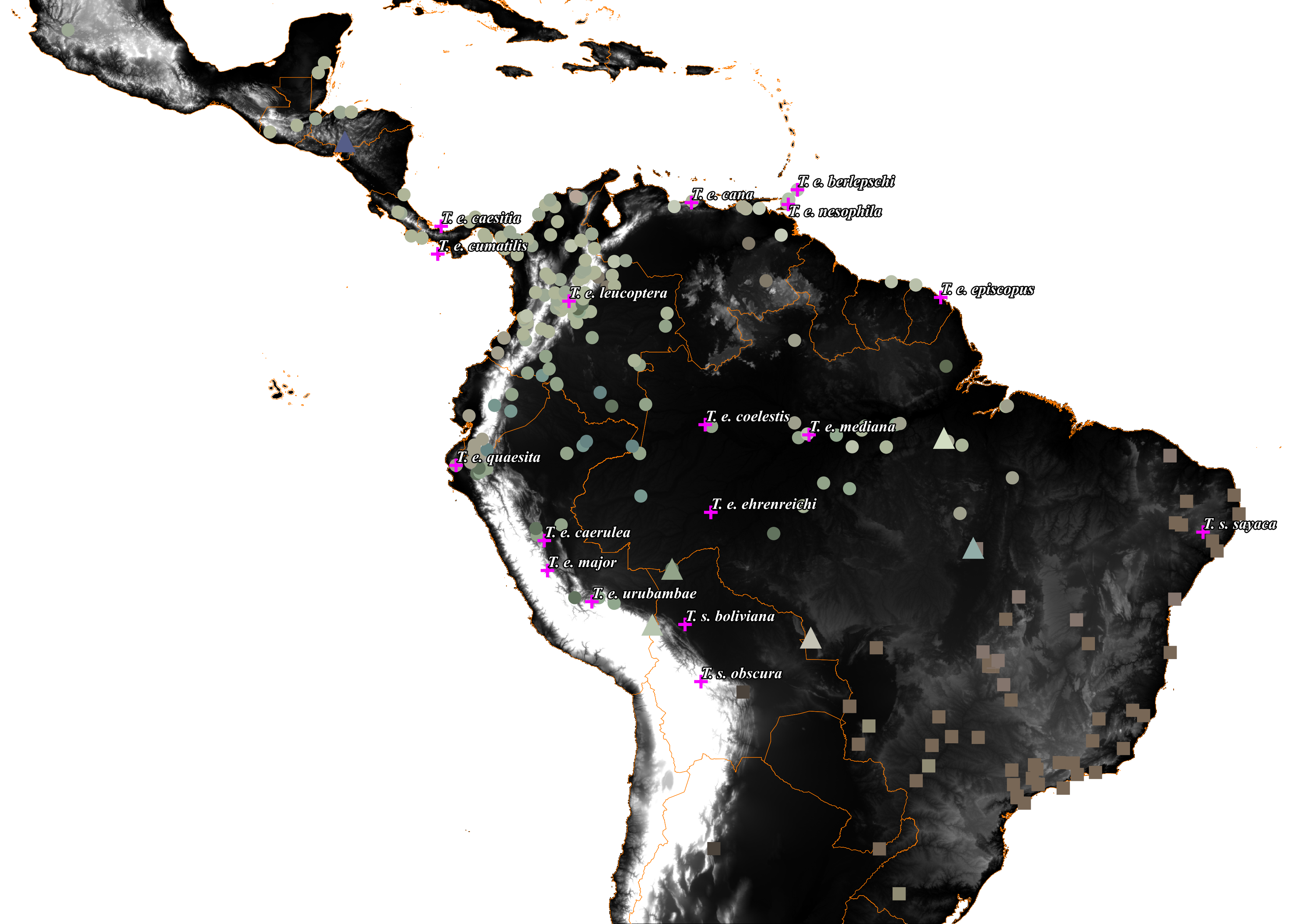

Supplement: S8 Fig — Each symbol represents a photographed specimen of T. episcopus (circles), T. sayaca (squares) or intermediate specimens (triangles). Colors of the symbols reflects the actual color of the crown, as extracted from photography with the HTML code. Pink crosses mark the type locality of each subspecies. Raw pictures available at figshare.com—S1 folder. (PNG) [file pone.0270892.s009.png]

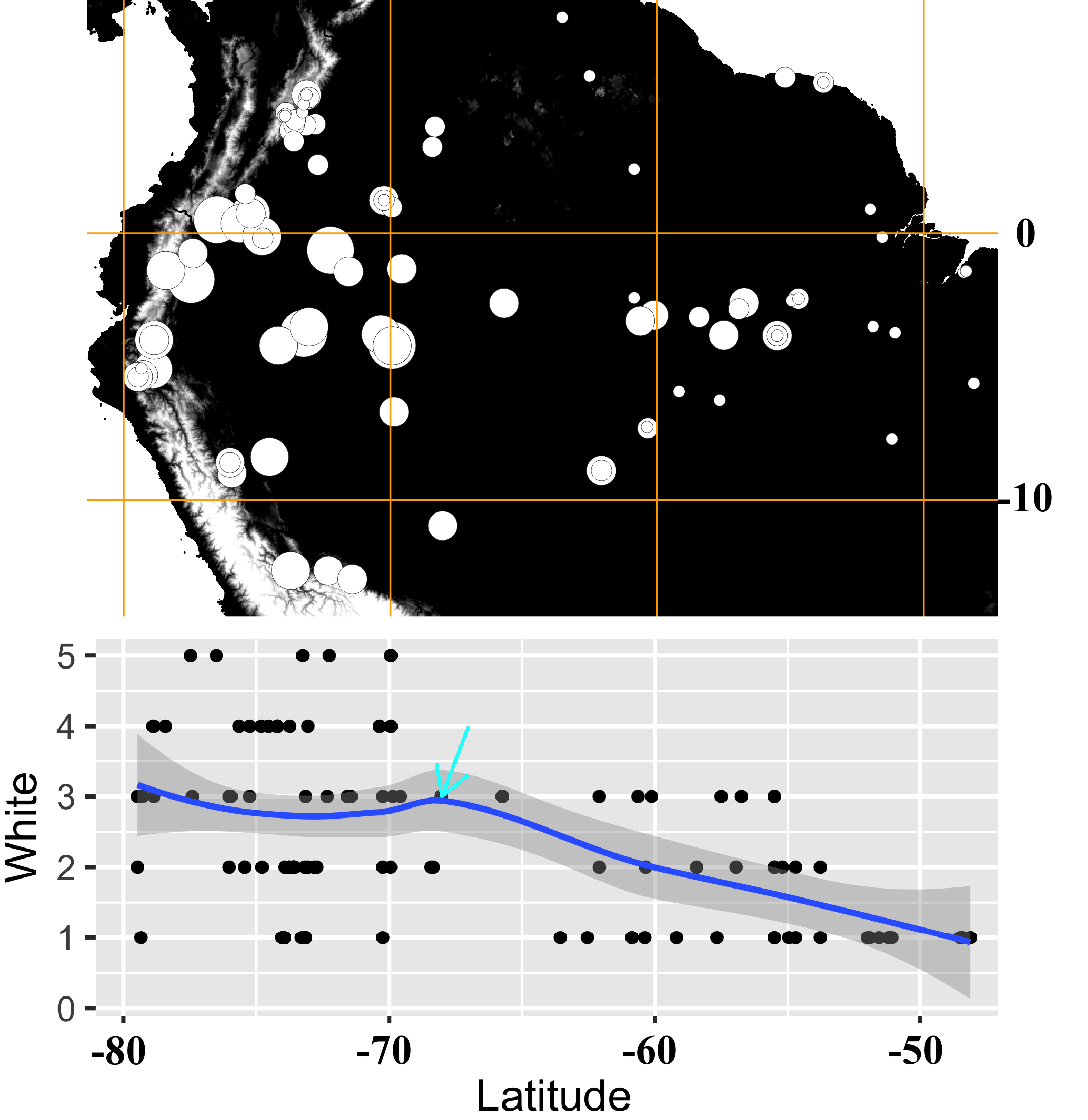

Supplement: S9 Fig — Each white circle represents a photographed specimen of T. episcopus. The amount of white was estimated using S1 Fig. The size of the with circles represent the amount of white on the wing greater coverts. Individuals with category 0 or no white are not on the map. Raw pictures available at figshare.com—S1 folder. (TIF) [file pone.0270892.s010.tif]

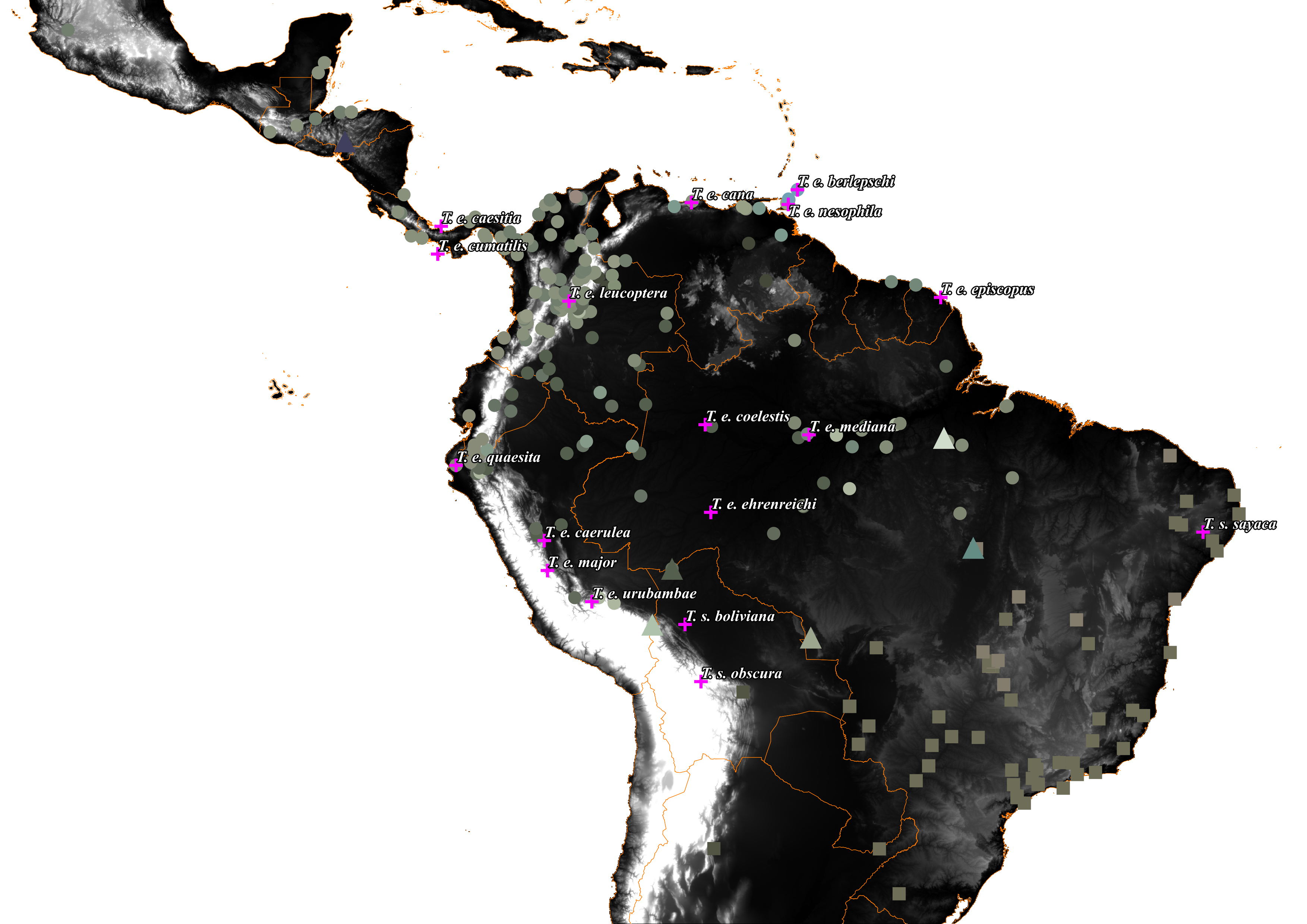

Supplement: S10 Fig — Each symbol represents a photographed specimen of T. episcopus (circles), T. sayaca (squares) or intermediate specimens (triangles). Colors of the symbols reflects the actual color of the back, as extracted from photography with the HTML code. Pink crosses mark the type locality of each subspecies. Raw pictures available at figshare.com—S1 folder. (PNG) [file pone.0270892.s011.png]

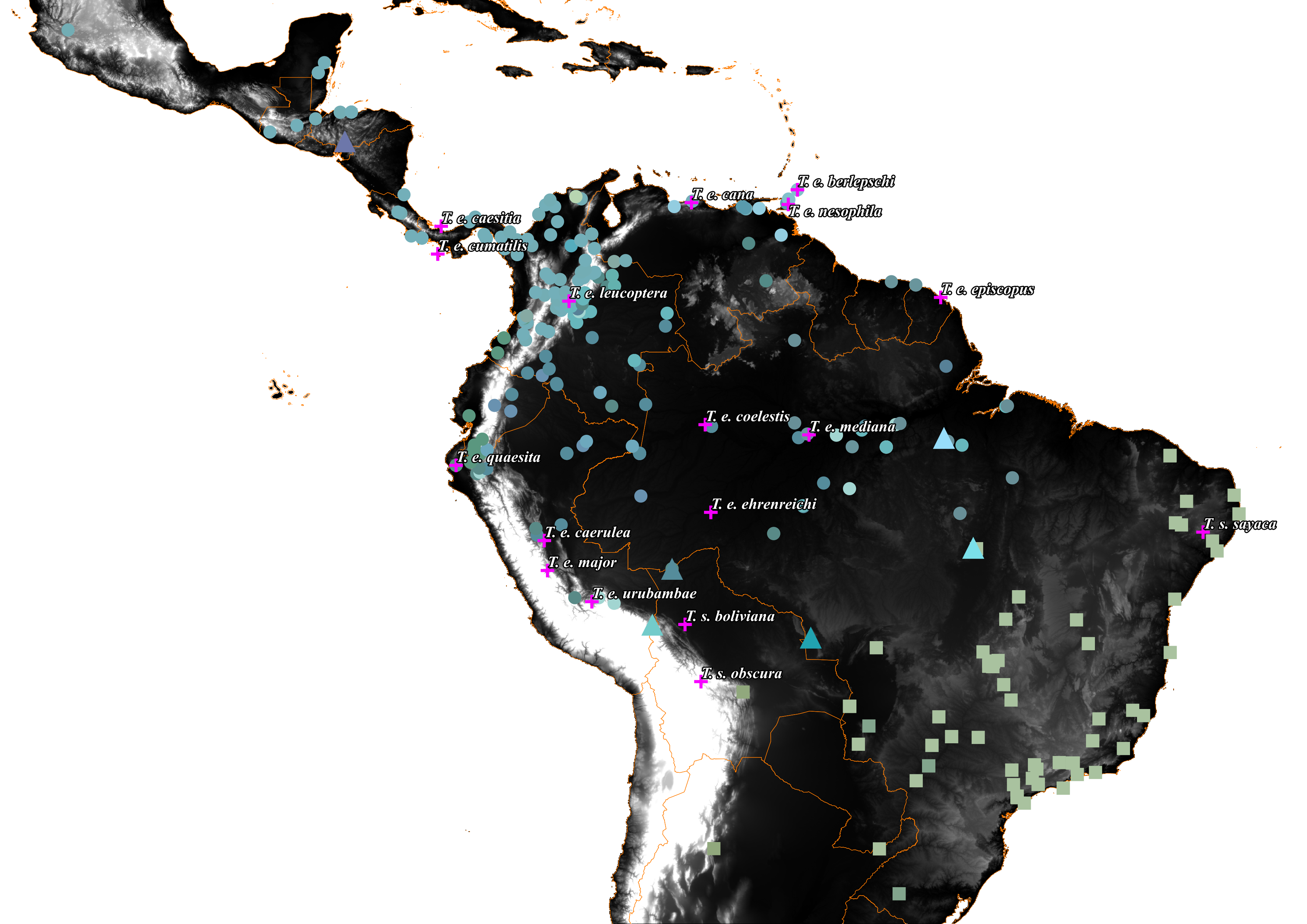

Supplement: S11 Fig — Each symbol represents a photographed specimen of T. episcopus (circles), T. sayaca (squares) or intermediate specimens (triangles). Colors of the symbols reflects the actual color of the primaries, as extracted from photography with the HTML code. Pink crosses mark the type locality of each subspecies. Raw pictures available at figshare.com—S1 folder. (PNG) [file pone.0270892.s012.png]

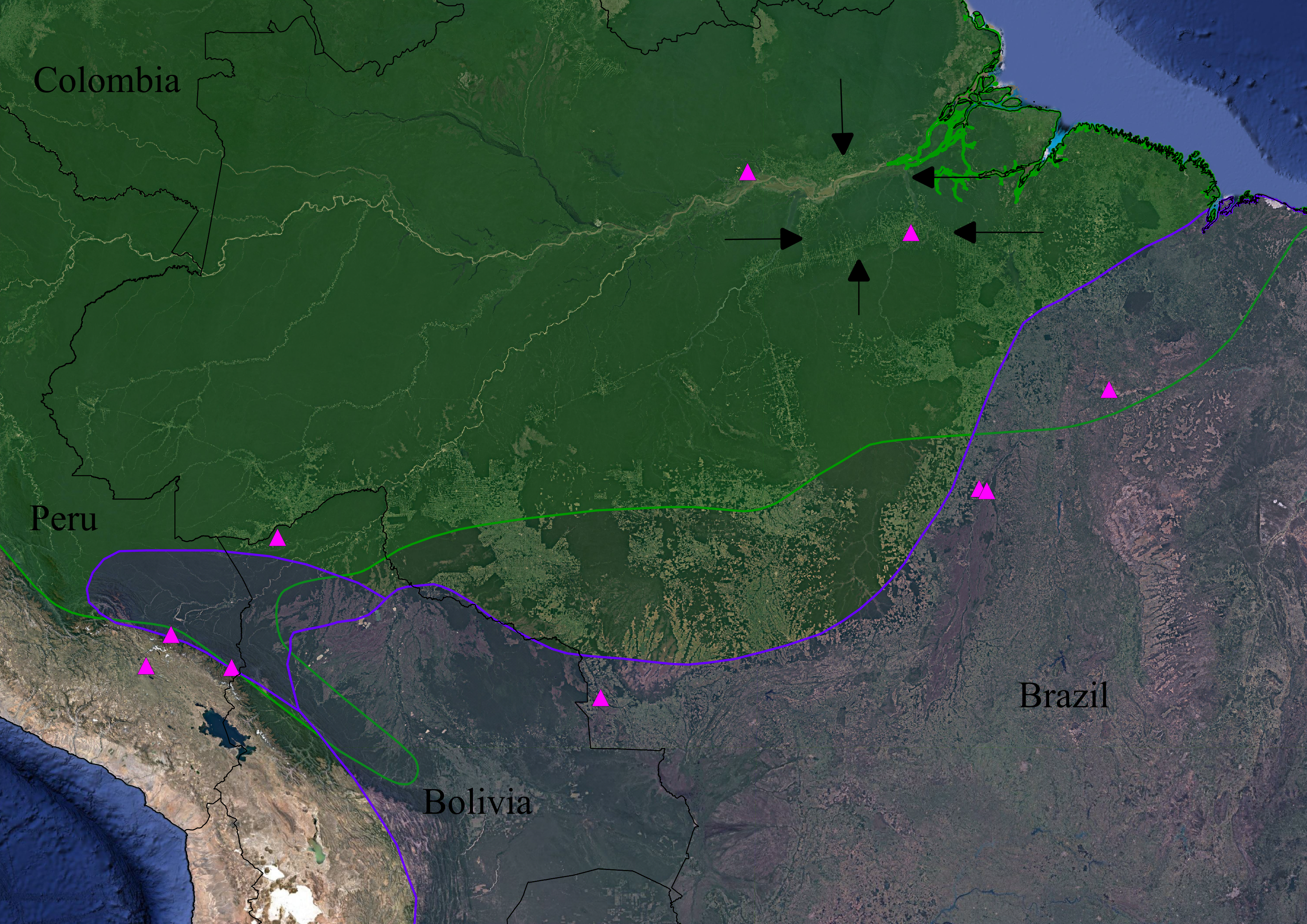

Supplement: S12 Fig — Pink triangles represent the locality of hybrid specimens in Table 1. Green and Purple represents the geographic distribution of T. episcopus and T. sayaca respectively [53, 54]. Black arrows indicate deforestation path that connect the Cerrado grasslands with the hybrid molecular specimens on the middle of the Amazon. Most of the highlighted deforestation path belongs to the Trans-Amazonian Highway. (PNG) [file pone.0270892.s013.png]

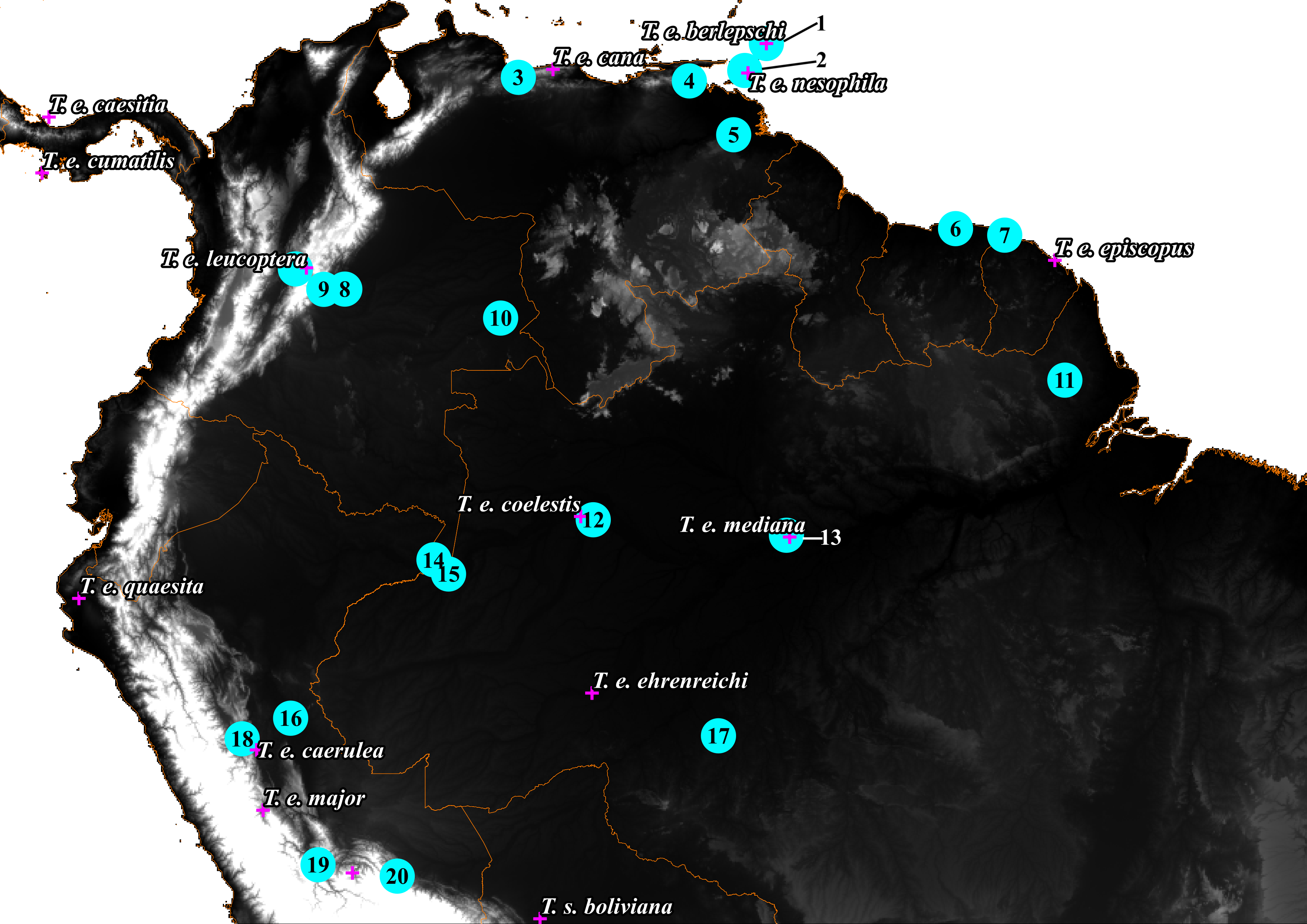

Supplement: S13 Fig — Pink crosses mark the type locality of each subspecies. Blue circles with numbers are link with the pictures on S1. (PNG) [file pone.0270892.s014.png]
